# Factors associated with access to sexual and reproductive health services among women with disabilities in Nepal

**DOI:** 10.1016/j.dialog.2022.100068

**Published:** 2022-10-30

**Authors:** Sampurna Kakchapati, Saugat Pratap KC, Santosh Giri, Sanju Bhattarai, Sushil Chandra Baral

**Affiliations:** HERD International, Bhaisepati, Nepal

**Keywords:** Women with disability, Sexual and reproductive health, Nepal

## Abstract

**Aim:**

The aim of the study is to investigate the relationships between social determinants and disability status and access and use of sexual and reproductive health services among women with disabilities in Nepal.

**Materials ad methods:**

This study used data on women with disability from the Multiple Indicator Cluster Survey (MICS) of 2019, in which 13,320 women and 290 women with disabilities were included for the analysis. We used bivariate analysis to compare the social determinants with disability status and multivariate logistic regression to determine the association between social determinants and access and use of sexual and reproductive health services among women with disabilities.

**Findings:**

The findings showed, in comparison with non-disabled women, women with disabilities had low education, low economic status, low media exposure and low access to sexual and reproductive health. On provincial level, those from Madesh [AOR = 0.22 (95%CI:0.06, 0.76)] and Lumbini [AOR = 0.24 (95%CI:0.06,0.88)] had lower attitude to violence. The usage of family planning (FP) methods among women with disabilities in Karnali [AOR = 3.57 (95% CI: 1.42–13.22)] and Sudurpashchim [AOR = 1.05 (95% CI: 1.01–1.071)] was higher than those in Province 1. Women with disabilities with secondary education were more than nine times [AOR = 9.28(95%CI:2.67,32,26)] and primary education had more than three times [AOR = 3.59 (95%CI:1.07, 12.02)] of knowledge on HIV/AIDS compared to those of no education. The odds of being tested for HIV/AIDS among women with disabilities with secondary education was more than eight times [AOR = 8.8 (95% CI:2.23–34.6)] than those of no education.

**Conclusion:**

This study provides noteworthy findings that women with disabilities have poor socioeconomic status, high-risk behavior, and low access to sexual and reproductive health services in Nepal. This study highlights the significance of actions needed to address sexual and reproductive health services in Nepal that unfairly impact women with disabilities.

## 1 Introduction

It is estimated that one billion people (one in every five women) worldwide have some form of disability, out of which 80% live in low resource settings. People with disabilities have poor educational attainment, low workforce participation, and limited access to health services and resources [1,2]. The discrimination and marginalization are such that many countries consider persons with disabilities as socially disadvantaged groups for targeted social and welfare programs [3,4]. In low-income countries such as Nepal, where gender-based discrimination is rife, women with disabilities experience the additional burden of exclusion with poorer health outcomes [4].

Sexual and reproductive health (SRH) is an essential part of health and sustainable development [2,5]. The SRH needs of the person with disabilities are comparable to others [6,7]; however, in many low-income countries, their SRH rights are neglected [4,6] due to popular belief that they don't have sexual drive [3,5]. In Nepal, studies highlight challenges related to health facilities and service providers- faced by a person with a disability in accessing SRH information and services. The barriers are greater for women with disabilities [4,6,8]. Determinants of SRH among women with disabilities are multifaceted [[5], [6], [7], [8]]. Low socioeconomic status reduces their ability to access health services; it is aggravated by limited availability and high cost of health services [8,9]. Even when SRH services are available, care and support received from health care providers and the attitude of the community towards women with disabilities are not optimal [10]. In addition, service providers lack appropriate equipment, educational materials, training to provide SRH services for women with disabilities [10,11]. Women with disabilities are two to four times more likely to experience emotional, physical, and sexual violence (including intimate partner violence, abuse by other family members, rape, forced sterilization, and/or abortion) than women without disability [12,13]. The vulnerability of women with disabilities to violence is compounded by economic dependency; social isolation; the perception that they will and/or cannot report violence and physically defend themselves; and their exclusion from violence prevention programs [10,13]. Violence against women with disabilities violates their SRH rights undermining their health and wellbeing [7,8,13].

Studies have reported that women with disabilities face challenges in health systems (service providers' attitudes and infrastructure), economic and societal challenges in accessing SRH services [10,14,15]. Several studies have explored the factors affecting access to and utilization of health services by persons with disabilities [8,9,14,15], however, fewer studies have explored these challenges among women with disabilities in Nepal. Therefore, this study aims to describe the relationships between disability status and access to SRH services. It also explores the relationship between social determinants (age, gender, wealth quintile, education, media use) and access to SRH services by women with disabilities.

## 2 Material and methods

We analyzed the data from Multiple Indicator Cluster Survey (MICS) [16], a nationally representative household survey conducted periodically in a number of developing countries including Nepal. The survey provides estimates on key maternal and child health indicators including SRH, nutrition, tobacco and alcohol use, and adult functioning across urban and rural areas of all the seven provinces of Nepal. A two-stage, stratified cluster sampling approach is used by MICS for sample selection. For this analysis, data from 13,320 adult women (18 years and above) was extracted from the MICS database to compare the distribution of social determinants, risk behaviors, access to SRH between women with and without disabilities. We further analyzed the data from 290 women with functional difficulties to explore the relationship between social determinants and access to SRH services.

### 2.1 Study variables

The MICS survey collected information on the adult functioning of the sampled women based on a “short set” of questions developed by the Washington Group on Disability Statistics (WG) reflecting six domains for measuring disability (seeing, hearing, walking, cognition, self-care, and communication) [26]. The response scales are measure in “No - no difficulty”, “Yes – some difficulty”, “Yes – a lot of difficulty” and “Cannot do at all”. If the respondent mentioned “Yes – a lot of difficulty” and “Cannot do at all” at least one of these six domains, it is counted as having functional disability. A binary variable for whether a person has a functional disability was created based on the 6-item module.

The outcome variables selected for this analysis are (1) attitude towards domestic violence: measured by asking if they think husbands or mothers-in-law are justified to hit or beat wives in a variety of situations; (2) use of family planning (FP) methods: measured by use of permanent and temporary family planning methods, (3) knowledge about HIV/AIDS: faithfulness to one's partner or avoiding multiple sex partners, and consistent and correct condom use or use of a condom during every sex act as HIV-preventive measures. Additionally, HIV related knowledge and misconceptions that a healthy-looking person can be infected with HIV, a person cannot get HIV from a mosquito bite or by sharing a meal with an HIV-infected person, and the respondent ever being tested on HIV/AIDS were also measured as outcome variables.

Similarly, based on the available literature [17,18], several individuals, households, and community-level characteristics were selected as independent variables. The individual-level characteristics were age, gender, education, marital status, number of children, use of media, smoking behavior, and alcohol consumption. Selected characteristics related to women such as menstrual hygiene management (MHM) defined as having a private place to wash and change at home and whether they used appropriate materials including reusable and non-reusable materials during last menstruation. Moreover, women characteristics such as ever receiving perinatal care during pregnancy, and place of last delivery (home delivery/ institutional delivery) were included in the analysis. Household-level characteristics included wealth index quintile (categorized as Poorest, Poorer, Middle, Richer, and Richest) which was calculated based on ownership of household amenities, facilities, assets, and access to health insurance. Lastly, the community level characteristics contained place of residence (rural or urban) and provinces.

### 2.2 Ethics considerations

As per the Statistical Act (1958), the study protocol for the Nepal MICS survey was approved by the Central Bureau of Statistics (CBS) along with UNICEF. Since this study involved the analyses of publicly available anonymized secondary data, ethical approval from respective institutions was not required. However, ethical approval for the survey was obtained from Central Bureau of Statistics (CBS).

### 2.3 Data analysis

The SPSS dataset available from MICS was imported to R statistical software for data analysis. The data was checked for completeness and inconsistencies. We compared the distribution of women with and without functional difficulties across demographic, socioeconomic, and behavioral characteristics. Pearson's chi-squared test for categorical and student's *t*-test for continuous variables was used to determine whether the differences in the characteristics were statistically significant between women with and without functional difficulties. A two-tailed *p*-value of <0.05 was considered to be statistically significant.

The variables that were statistically significant were then fitted into logistic regression models, constructed only among a subset of women with functional difficulties. Initially, explanatory determinants were included in the model one at a time to examine their univariate relationship with the outcome. Multivariate logistic regression models were then used to identify the most important determinants for each outcome. We estimated an unadjusted odds ratio for the independent variables against attitude towards violence, current use of family planning methods, knowledge and misconceptions on HIV/AIDS, and ever being tested for HIV/AIDS. Further, adjusted odds ratios were estimated after controlling for the place of residence, province, education, wealth quintile, health insurance, use of media, smoking behavior, and alcohol consumption. The results are presented in a combined table with the significant results highlighted.

## 3 Results

Table 1 shows the comparison of social determinants and SRH behaviors among women with and without disabilities. There was a significant association between province, age, education, marital status, wealth quantile, use of media, cigarette smoking, private place for menstrual hygiene management, ever heard about HIV/AIDs, knowledge on HIV/AIDs with functional disabilities. Bagmati province had the highest proportion of women with (22.4%) and without (20.1%) disabilities. Compared to women without disabilities, the mean age and number of children were higher among women with disabilities. Proportion of women with no education (52.1% vs 30.3%), and women belonging to poorest wealth quintile (32.8% vs 23.3%) was also observed to be higher among women with disabilities. Similarly, a higher proportion of women with disabilities had ever smoked (23.4% vs 8.2%). In addition, the use of at least one of the listed media (55.5% vs 62.7%), menstrual hygiene management (72.8% vs 83.9%) was lower among the women with disabilities. Likewise, perinatal care (7.9% vs 18%), heard about HIV/AIDs (60.7% vs 73.4%), and knowledge on HIV/AIDs (12.1% vs 197%) were comparatively lower among women with disabilities compared to women without disabilities.

**Table 1:** Comparison of social determinants, risk behaviors and sexual and health behaviors among women with and without disabilities.

Characteristics	Functional Disabilities			Test Statistics (df)	P-value
	Yes	No	Total		
	290	13,030	13,320		
Area				1.29(1)	0.255
Urban	161 (55.5)	7667 (58.8)	7828 (58.8)		
Rural	129 (44.5)	5363 (41.2)	5492 (41.2)		
Province				63.13(6)	< 0.001
Province 1	31 (10.7)	1817 (13.9)	1848 (13.9)		
Madesh	30 (10.3)	1973 (15.1)	2003 (15)		
Bagmati	65 (22.4)	2618 (20.1)	2683 (20.1)		
Gandaki	47 (16.2)	1473 (11.3)	1520 (11.4)		
Lumbini	31 (10.7)	2162 (16.6)	2193 (16.5)		
Karnali	64 (22.1)	1332 (10.2)	1396 (10.5)		
Sudharpashchim	22 (7.6)	1655 (12.7)	1677 (12.6)		
Age				11.3(13318)	< 0.001
Mean(SD)	36.5 (9.1)	30.7 (8.7)	30.8 (8.7)		
Education				73.27(3)	< 0.001
None	151 (52.1)	3943 (30.3)	4094 (30.7)		
Primary	75 (25.9)	3609 (27.7)	3684 (27.7)		
Secondary	57 (19.7)	4368 (33.5)	4425 (33.2)		
Higher Secondary	7 (2.4)	1110 (8.5)	1117 (8.4)		
Married				6.52 (2)	0.038
Currently married	239 (82.4)	11,286 (86.6)	11,525 (86.5)		
Formerly married	12 (4.1)	289 (2.2)	301 (2.3)		
Never married	39 (13.4)	1455 (11.2)	1494 (11.2)		
Children				6.68(13318)	< 0.001
Mean(SD)	2.5 (1.8)	1.9 (1.5)	1.9 (1.5)		
Wealth Quintile				16.91 (4)	0.002
Poorest	95 (32.8)	3038 (23.3)	3133 (23.5)		
Poorer	63 (21.7)	2660 (20.4)	2723 (20.4)		
Middle	49 (16.9)	2646 (20.3)	2695 (20.2)		
Richer	47 (16.2)	2625 (20.1)	2672 (20.1)		
Richest	36 (12.4)	2061 (15.8)	2097 (15.7)		
Health Insurance				1.13 (1)	0.287
Yes	21 (7.2)	751 (5.8)	772 (5.8)		
No	269 (92.8)	12,279 (94.2)	12,548 (94.2)		
Use of three media				1.22 (1)	0.269
Yes	7 (2.4)	474 (3.6)	481 (3.6)		
No	283 (97.6)	12,556 (96.4)	12,839 (96.4)		
Use of one media				6.26(1)	0.012
Yes	161 (55.5)	8171 (62.7)	8332 (62.6)		
No	129 (44.5)	4859 (37.3)	4988 (37.4)		
Ever Cigarette Smoking				84.44(1)	< 0.001
Yes	68 (23.4)	1069 (8.2)	1137 (8.5)		
No	222 (76.6)	11,961 (91.8)	12,183 (91.5)		
Current Cigarette Smoking				0.02(1)	0.886
Yes	35 (58.3)	563 (57.4)	598 (57.4)		
	25 (41.7)	418 (42.6)	443 (42.6)		
Alcohol Consumption				2.49(1)	0.115
Yes	70 (24.1)	2653 (20.4)	2723 (20.4)		
No	220 (75.9)	10,377 (79.6)	10,597 (79.6)		
Menstrual Hygiene Management				4.98(1)	<0.001
Yes	211 (72.8)	10,935 (83.9)	11,146 (83.7)		
No	79(27.2)	2095 (16.1)	2174 (16.3)		
Current using Family Planning				0.21(1)	0.648
Yes	120 (41.4)	5257 (40.3)	5377 (40.4)		
No	170 (58.6)	7773 (59.7)	7943 (59.6)		
Received Perinatal Care				19.5(1)	<0.001
Yes	23 (7.9)	2340 (18)	2363 (17.7)		
No	267 (92.1)	10,690 (82)	10,957 (82.3)		
Place of Delivery of last child				137(1)	0.06
Home	5 (1.7)	547 (4.2)	552 (4.1)		
Health Institution	285 (98.3)	12,483 (95.8)	12,768 (95.9)		
Attitude towards domestic Violence				0.13(1)	0.714
Positive	86 (29.7)	3995 (30.7)	4081 (30.6)		
Negative	204 (70.3)	9035 (69.3)	9239 (69.4)		
Hear about HIV				24.4(1)	< 0.001
Yes	176 (60.7)	9597 (73.7)	9773 (73.4)		
No	114 (39.3)	3433 (26.3)	3547 (26.6)		
Knowledge on HIV				4.11(1)	0.001
Yes	35 (12.1)	2562 (19.7)	2597 (19.5)		
No	225 (87.9)	10,468 (80.3)	10,723 (80.5)		
Tested for HIV				0.07(1)	0.3
Yes	22 (7.5)	1222 (9.4)	1244 (13.8)		
No	268 (92.5)	11,808 (90.6)	12,076 (90.7)		

Factors associated with attitude on violence and current use of FP methods among women with disabilities are presented in Table 2. In the adjusted model, Madhesh [AOR = 0.22 (95% CI: 0.06, 0.76)] and Lumbini province [AOR = 0.24 (95%CI: 0.06, 0.88)] had significantly lower odds of attitude on domestic violence. Inversely, compared to Province 1, the odds for the current use of FP methods was more than four times [AOR = 4.6 (95% CI: 1.6, 13.22)] for Karnali Province. Women belonging to the poor wealth quintile had more than two times [AOR = 2.18 (95% CI: 1.0, 4.78)] odds of using FP methods compared to those in the poorest wealth quintile.

**Table 2:** Factors associated with attitude towards domestic violence and current use of FP methods among women with disabilities of Nepal.

Variable	Attitude of Domestic Violence			Current Use of FP Methods		
	N(%)	COR(95%CI)	AOR(95%CI)	N(%)	COR(95%CI)	AOR(95%CI)
All	86 out of 290 (29.7%)			120 out of 290 (41.4%)		
Area						
Urban	44 (27.3)	Ref		56 (34.8)	Ref	Ref
Rural	42 (32.6)	1.28 (0.77,2.13)		64 (49.6)	1.85 (1.15,2.96)*	1.64 (0.93,2.91)
Province						
Province 1	13 (41.9)	Ref	Ref		Ref	Ref
Madesh	5 (16.7)	0.28 (0.08,0.92)*	0.22(0.06, 0.76)*	9 (29)	1.42 (0.48,4.14)	1.21 (0.39,3.76)
Bagmati	28 (43.1)	1.05 (0.44,2.49)	1.49(0.59, 3.77)	11 (36.7)	1.53 (0.61,3.84)	1.75 (0.63,4.82)
Gandaki	14 (29.8)	0.59 (0.23,1.52)	0.60(0.22, 1.59)	25 (38.5)	1.52 (0.57,4.02)	1.44 (0.51,4.05)
Lumbini	4 (12.9)	0.21 (0.06,0.73)*	0.24(0.06, 0.88)*	18 (38.3)	1.34 (0.46,3.92)	1.56 (0.5,4.81)
Karnali	12 (18.8)	0.32 (0.12,0.83)*	0.38(0.13,1.09)	11 (35.5)	3.57 (1.42,8.98)*	4.6 (1.6,13.22)*
Sudharpashchim	10 (45.5)	1.15 (0.38,3.47)	1.06(0.34, 3.30)	38 (59.4)	1.4 (0.44,4.48)	1.54 (0.45,5.26)
Age	37 (9.5)	1.01 (0.98,1.04)		38.6 (7.3)	1.05 (1.02,1.08)*	1.04 (0.98,1.07)
Education						
Illiterate	48 (31.8)	Ref		73 (48.3)	Ref	
Primary	23 (30.7)	0.95 (0.52,1.73)		27 (36)	0.6 (0.34,1.06)	
Secondary and above	15 (23.4)	0.66 (0.34,1.29)		20 (31.2)	0.49 (0.26,0.9)	
Wealth Quantile						
Poorest	22 (23.2)	Ref	Ref	45 (47.4)	Ref	Ref
Poorer	25 (39.7)	2.18 (1.09,4.37)	1.89(0.85, 4.22)	34 (54)	1.3 (0.69,2.47)	2.18 (1.0,4.78)*
	20 (40.8)	2.29 (1.09,4.81)	2.09(0.87, 5.02)	16 (32.7)	0.54 (0.26,1.11)	1.18 (0.49,2.85)
Richer	12 (25.5)	1.14 (0.51,2.56)	0.74(0.29, 1.88)	13 (27.7)	0.42 (0.2,0.9)	1.05 (0.42,2.64)
Richest	7 (19.4)	0.8 (0.31,2.08)	0.47(0.15, 1.42)	12 (33.3)	0.56 (0.25,1.24)	1.36 (0.49,3.75)
Use of Media						
No	35 (27.1)	Ref		54 (41.9)	Ref	
Yes	51 (31.7)	1.25 (0.75,2.08)		66 (41)	0.96 (0.6,1.54)	
Health Insurance						
No	81 (30.1)	Ref		114 (42.4)	Ref	
Yes	5 (23.8)	0.73 (0.26,2.05)		6 (28.6)	0.54 (0.2,1.44)	
Smoking Behaviors						
No	65 (29.3)	Ref		83 (37.4)	Ref	
	21 (30.9)	1.08 (0.6,1.95)		37 (54.4)	2 (1.15,3.46)*	1.36 (0.72,2.56)
Alcohol Consumption						
No	64 (29.1)	Ref		87 (39.5)	Ref	
Yes	22 (31.4)	1.12 (0.62,2)		33 (47.1)	1.36 (0.79,2.34)	

COR = Crude Odds Ratio, AOR = Adjusted Odds Ratio, * significant p-value<0.005.

Table 3 shows that 12.1% of women with disabilities had knowledge about HIV/AIDS and 7.5% had tested for HIV/AIDs. We found education was significantly associated with knowledge and misconceptions about HIV/AIDs. Women with education level of secondary and above [AOR = 9.28 (95% CI: 2.67, 32.3)], and primary level [AOR = 3.59 (95% CI: 1.07, 12.02)] had higher odds of having knowledge on HIV/AIDS compared to those who were not literate. Similarly, the odds of being tested for HIV/AIDS was more than eight times higher [AOR = 8.8 (95% CI: 2.23, 34.6)] for those with secondary education compared to those without.

**Table 3:** Factors associated with Knowledge on HIV/AIDS and Tested for HIV/AIDS among women with disabilities of Nepal.

Variable	Knowledge on HIV/AIDS			Tested for HIV/AIDS		
	N(%)	COR(95%CI)	AOR(95%CI)	N(%)	COR(95%CI)	AOR(95%CI)
All	35 out of 290 (12.1%)			35 out of 290 (7.5%)		
Area						
Urban	27 (16.8)	Ref	Ref	14 (8.7)	Ref	
Rural	8 (6.2)	0.33 (0.14,0.75)*	0.58 (0.22,1.57)	8 (6.2)	0.69 (0.28,1.71)	
Province						
Province 1	5 (16.1)	Ref		2 (6.5)	Ref	
Madesh	1 (3.3)	0.18 (0.02,1.64)		0 (0)	1.33 (0.27,3.5)	
Bagmati	13 (20)	1.3 (0.42,4.04)		9 (13.8)	2.33 (0.47,11.5)	
Gandaki	6 (12.8)	0.76 (0.21,2.75)		1 (2.1)	0.32 (0.03,3.63)	
Lumbini	3 (9.7)	0.56 (0.12,2.57)		4 (12.9)	2.15 (0.36,12.6)	
Karnali	6 (9.4)	0.54 (0.15,1.92)		2 (3.1)	0.47 (0.06,3.49)	
Sudharpashchim	1 (4.5)	0.25 (0.03,2.29)		4 (18.2)	3.2 (0.53,19.42)	
Age	33.6 (8.1)	0.96(0.92,0.99)*	1.01 (0.96,1.05)	34.2 (7.7)	0.97 (0.93,1.02)	
Education						
Illiterate	5 (3.3)	Ref	Ref	3 (2)	Ref	
Primary	10 (13.3)	4.49 (1.48,13.67)*	3.59 (1.07,12.1)*	6 (8)	4.29 (1.04,17.6)*	3.16 (0.73,13.6)
Secondary and above	20 (31.2)	13.27 (4.71,37.4)*	9.28 (2.67,32.3)*	13 (20.3)	12.6(3.44,45.9)*	8.8 (2.23,34.6)*
Wealth Quantile						
Poorest	4 (4.2)	Ref		3 (3.2)	Ref	Ref
Second	6 (9.5)	2.39 (0.65,8.85)	2.13 (0.51,8.89)	2 (3.2)	1.01 (0.16,6.19)	0.93 (0.15,5.9)
Middle	6 (12.2)	3.17 (0.85,11.84)	1.5 (0.36,6.34)	5 (10.2)	3.48 (0.8,15.24)	2.24 (0.48,10.3)
Fourth	8 (17)	4.67 (1.33,16.41)*	1.84 (0.45,7.43)	6 (12.8)	4.49 (1.07,18.8)	2.63 (0.59,11.8)
Richest	11 (30.6)	10.1 (2.94,34.14)*	2.29 (0.55,9.56)	6 (16.7)	6.13 (1.44,26.1)	2.39 (0.51,11.1)
Use of Media						
No	6 (4.7)	Ref	Ref	8 (6.2)	Ref	
	29 (18)	4.5 (1.81,11.22)*	1.9 (0.67,5.38)	14 (8.7)	1.44 (0.58,3.55)	
Health Insurance						
No	32 (11.9)	Ref		20 (7.4)	Ref	
Yes	3 (14.3)	1.23 (0.34,4.43)		2 (9.5)	1.31 (0.28,6.03)	
Smoking Behaviors						
No	29 (13.1)	Ref		18 (8.1)	Ref	
Yes	6 (8.8)	0.64 (0.26,1.62)		4 (5.9)	0.71 (0.23,2.17)	
Alcohol Consumption						
No	28 (12.7)	Ref		18 (8.2)	Ref	
Yes	7 (10)	0.76 (0.32,1.83)		4 (5.7)	0.68 (0.22,2.08)	

COR = Crude Odds Ratio, AOR = Adjusted Odds Ratio, * means p-value<0.005.

## 4 Discussion

This study examined the relationship between disability status, and access to and use of SRH services among women with disabilities in Nepal. We found a significant difference in education wealth quantile, smoking behaviors, menstrual hygiene management (MHM) between women with and without disabilities.

Findings consistent with several other previous studies [8,9,13,15,17] from low-income countries were revealed in this study, where lower education and economic status were attributed to women with disabilities. In fact, poverty and disability are inextricably linked; [12,19] disabilities are ‘both cause and consequence of poverty’, reinforcing each other to increase vulnerability and exclusion. Disability adversely affects socio-economic opportunities. Persons with disabilities are likely to have lower educational attainment, employment opportunities and income than the rest of the population [17,18]. Similarly, women with disabilities have higher rates of unemployment and lower-paying jobs [12,19]. Women with disabilities were also more likely to be smokers, aligning with previous findings reporting significantly higher smoking habits among adults with disabilities [20,21]. A recent STEP survey in 2020 reported socioeconomic status such as low levels of educational achievement, unemployment and poverty as major determinants of tobacco use and smoking cessation rates [21]. Likewise, women who smoked had a significantly higher risk of infertility and pregnancy complications [22]. The higher rates of smoking among people with disabilities might be due to poor economic status, living in a restrictive environment, and high levels of stress, and anxiety arising from having to live with disability [17,23]. These findings suggest future SRH interventions to focus on specific “socio-economic factors” for the specific type of disability to improve access and use of those services by women/persons with disabilities.

Our findings show that media use was lower among women with disabilities; similar to other studies that reported social media uses among person with disabilities was moderately low [9,24]. Fear and stereotypes about disability are deeply ingrained in our culture and it is reflected in media coverage as well [24]. Moreover, presence of disability-friendly programs on the media are limited, which can be attributed to low media coverage among women with disabilities [24]. A low level of media use among women with disabilities is likely to reduce access to SRH messages and information. According to the Nepal Demographic Health Survey, more than one-third (37%) of women reported their sources of SRH information was through media [25]. These factors, in combination, pose greater risk to menstrual hygiene among women with disabilities. Women with disabilities were found to be unable to maintain their menstrual hygiene properly at varying levels. Moreover, persons with disabilities are considered ‘dirty’ and contagious, and thus, often not allowed to use public latrines and water points [15,26]. Inaccessibility to latrines goes in tandem with availability of disability friendly latrines and menstrual hygiene products. One systematic review reported that women with disabilities had low levels of satisfaction with the menstrual product as they found them uncomfortable [26,27] highlighting the need of training and support in MHM for women with disabilities and their caretakers. In Nepal, during the mensuration period, women are considered as impurity and are not allowed to stay in the family [28]. In the rural parts of Karnali and Sudurpashchim province, women/girls are even banished to sheds during their menstrual period (called *chhaupadi)*. Embedded in the cultural beliefs, menstrual restrictions including *chhaupadi* are generally considered a purity practice by the local community [29]. Disability is viewed as a curse, in the eyes of society, women with disabilities not complying with menstrual restrictions are doubly cursed epitomizing the layers of discrimination faced by women with disabilities [14,27].

Women with disabilities were more likely to delay or have inadequate prenatal care, and have postpartum check-ups. These delays could partially be attributed to the negative experiences of women with disabilities with their health care providers [10,25]. Women with disabilities often report that their health care providers could not manage their pregnancies effectively, possess negative stereotypes about their sexuality, disapprove of their pregnancy, and question their ability to parent [6]. A study documenting experiences of women with disabilities in using maternal health services in Nepal reported poor knowledge, skills, and negative attitudes of health care providers towards women with disabilities [6,13]. Lower levels of knowledge on HIV were also observed among women with disabilities. Studies from countries in Africa indicate that person with disabilities are less likely to access information about HIV prevention [30]. This may due to low level of education and income among persons with disabilities compared to those without [30,31]. Women with disabilities also encounter sexual violence [13,16,25] more often than others, and the lower level of education deprives them from accessing HIV knowledge, consequently, increasing their risk of HIV infection and lowering their chances of seeking HIV testing services. In fact, persons with disabilities are routinely left out of HIV/AIDS outreach efforts because many healthcare professionals falsely assume them to be at lower risk [28,29]. This has been a major barrier for inclusion of person with disabilities in HIV/AIDS outreach efforts in many countries including Nepal [30,31].

The negative attitude on domestic violence among women with disabilities in Lumbini and Madhesh province was significantly higher compared to province 1. Dowry-related domestic violence, polygamy, dowry, beatings, rape are common in these provinces and the women victims do not get justice easily [5,29,32]. In the recent years the federal and provincial level have come together to tackle these issues such self-defense training at schools, awareness programs to reduce violence and crimes against women. These factors may the reasons for high negative attitude on domestic violence among women with disabilities in the study.

In terms of utilization of family planning services, this study found that women with disabilities from Karnali province had higher chances of adopting family planning methods compared to Province 1 coherent with findings from studies [16,25,30]. Karnali province has comparably higher fertility rates and the child marriage is common in these provinces. In the recent years, there were extensive campaign to raise awareness about family planning in the Karnali province as the family planning services are available through government, social marketing and private health facilities and available free of cost in public health facilities [25,33].

This study, to our knowledge, is the first one to explore factors associated with SRH access among women with disabilities that has generated generalizable findings based the data from a nationally representative population survey. However, the cross-sectional nature of the data collection also limits this study from attempting to establish a causal association. Furthermore, the use of existing data for the analysis also limited our ability to devise more sensitive variables to measure access to SRH services. And, subgroup analysis by a different type of disability was not possible.

## 5 Conclusions

In public discourses, policies, programming, and services, the sexual and reproductive health of women with disabilities are largely overlooked. This is largely due to a lack of understanding that they too have sexual and reproductive desires, goals, and hopes. Women with disabilities are marginalized and they experience health disparities compared to their counterparts. Their access to quality health care services including SRH is poor, likely to have unmet sexual and reproductive health needs. There is an urgent need for focused studies to determine barriers and facilitators faced by women with disabilities to develop disability-friendly SRH services. This will help in recognizing that women with disabilities are sexual beings and should not be pathologised. Rather policymaking and programming should be rights-based, gender responsive and disability inclusive.

## Declaration of Competing Interest

The authors declared no potential conflicts of interest with respect to the research, authorship, and/or publication of this article.
